# Supplementary figures and images for: Inhibition of Connexin 43 reverses ox-LDL-mediated inhibition of autophagy in VSMC by inhibiting the PI3K/Akt/mTOR signaling pathway
Source: PeerJ. 2022 Mar 16;10:e12969. doi: 10.7717/peerj.12969 (PMC8934045; doi:10.7717/peerj.12969)

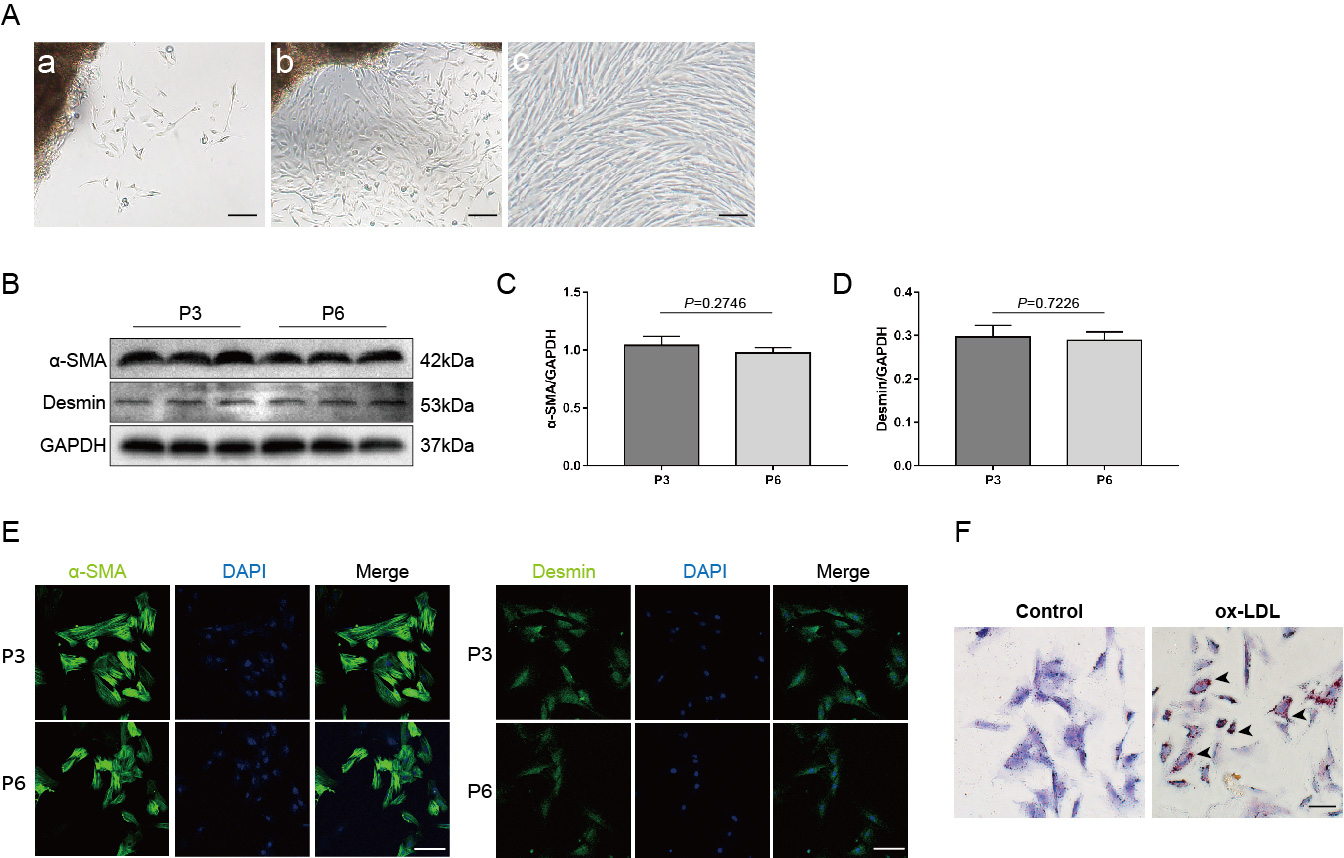

Supplement: Supplemental Information 1 — (A) Growth of SMCs. (a) At 4–7 days of culture, the cells climbed out from the sides of the blocks. (b) On the 15th day of culture, the cells covered the bottom of the dish. (c) The cells exhibited typical peak-valley growth patterns; scale bar: 100 μm. (B) The expression of α-SMA and Desmin on P3 and P6 VSMCs. (C and D) Statistical analysis of α-SMA and Desmin protein expression (n = 3, P = 0.2746 and 0.7226). (E) Expression and localization of α-SMA and Desmin in P3 and P6 VSMCs. Nuclei were counterstained with DAPI (blue). Scale bar: 50 μm. (F) Identification of the FC model. After ox-LDL intervention, the number of oil red O-positive cells in primary cultured VSMCs was significantly higher than that in the control group. The black arrow indicates the red-stained lipid droplets in the cytoplasm. Scale bar: 100 μm. [file peerj-10-12969-s001.jpg]

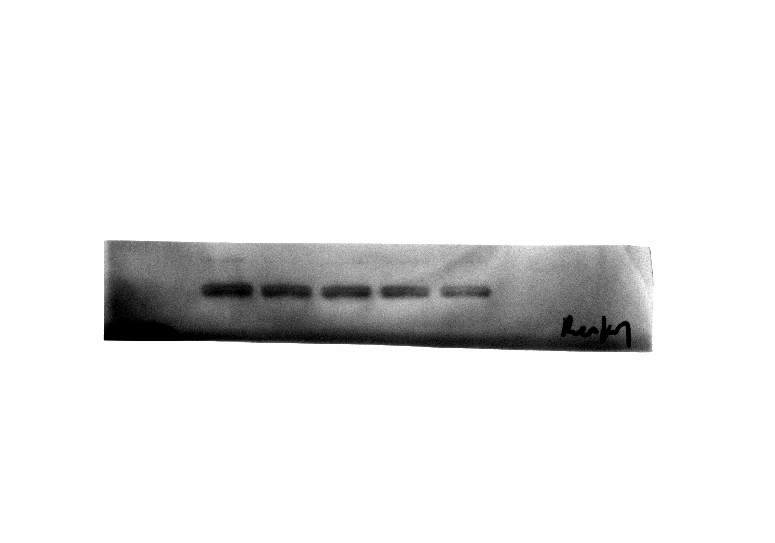

Supplement: Supplemental Information 3 [file peerj-10-12969-s003.zip › Initial data/FIG 1/Dose Beclin 1.tif]

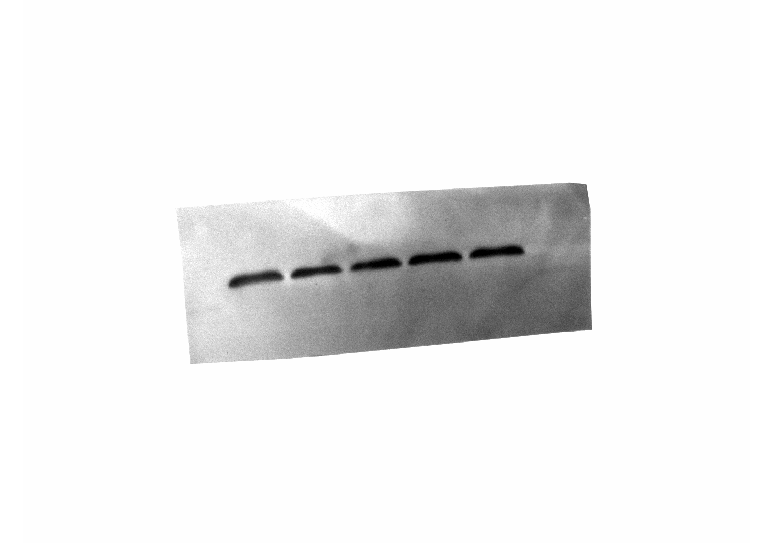

Supplement: Supplemental Information 3 [file peerj-10-12969-s003.zip › Initial data/FIG 1/Dose GAPDH.tif]

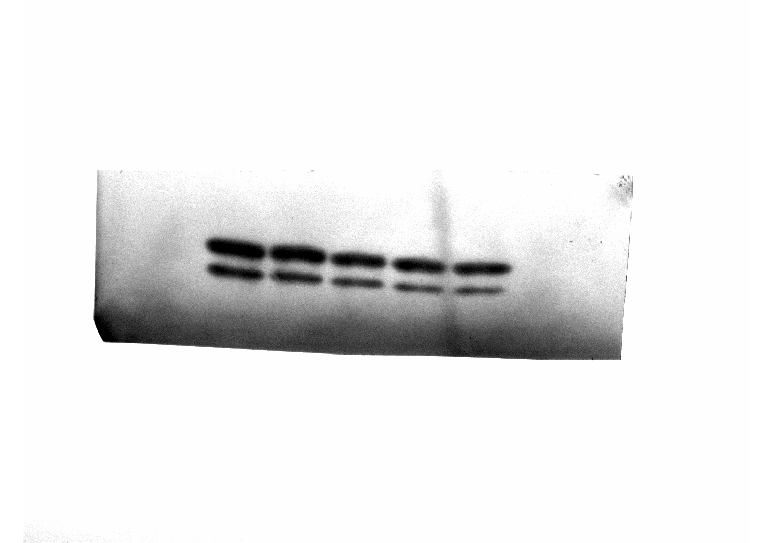

Supplement: Supplemental Information 3 [file peerj-10-12969-s003.zip › Initial data/FIG 1/Dose LC3B.tif]

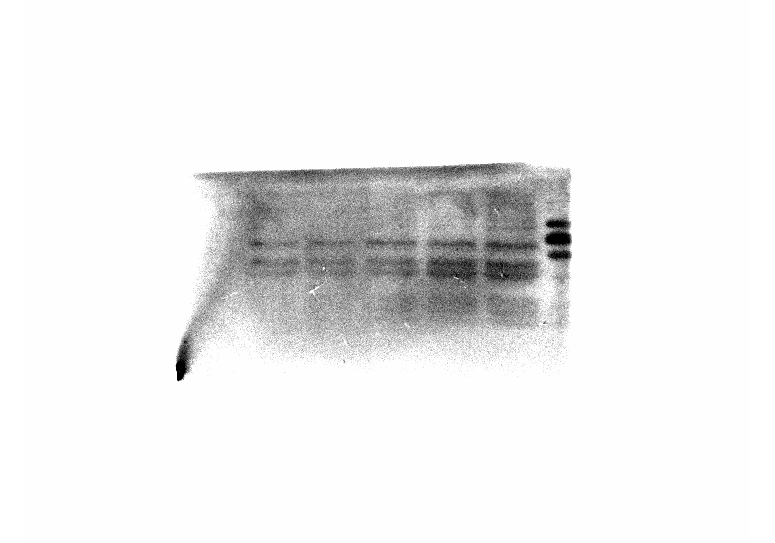

Supplement: Supplemental Information 3 [file peerj-10-12969-s003.zip › Initial data/FIG 1/Dose p62.tif]

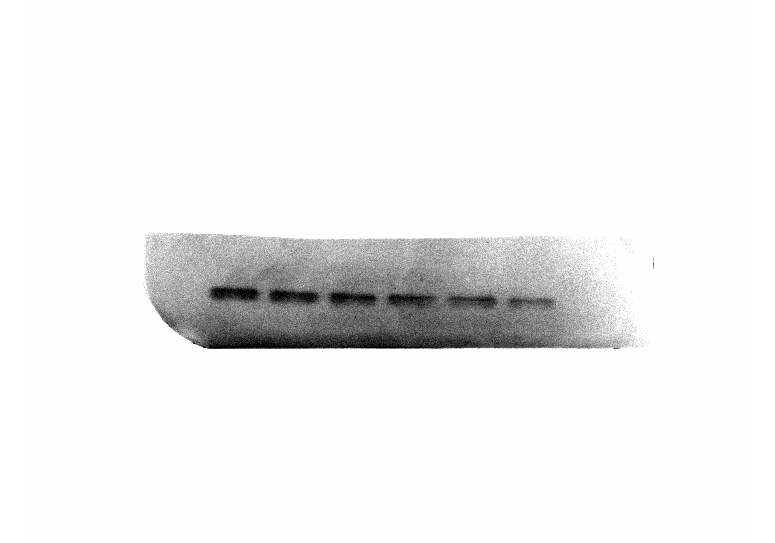

Supplement: Supplemental Information 3 [file peerj-10-12969-s003.zip › Initial data/FIG 1/Time Beclin 1.tif]

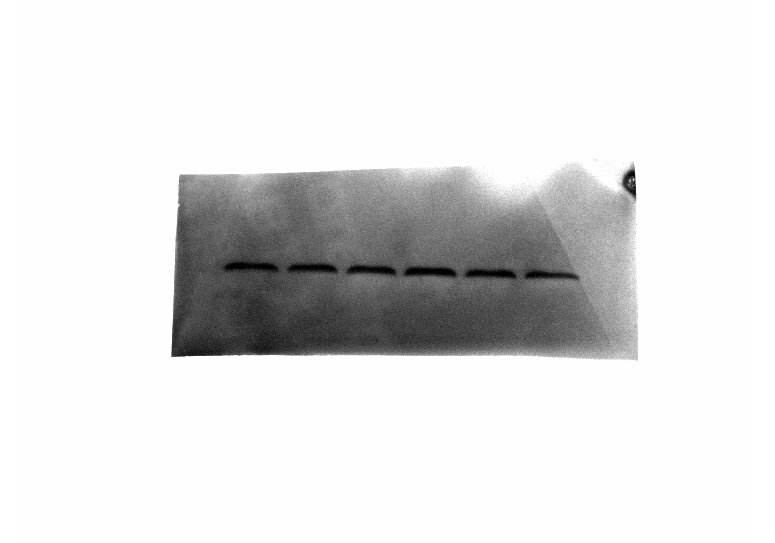

Supplement: Supplemental Information 3 [file peerj-10-12969-s003.zip › Initial data/FIG 1/Time GAPDH.tif]

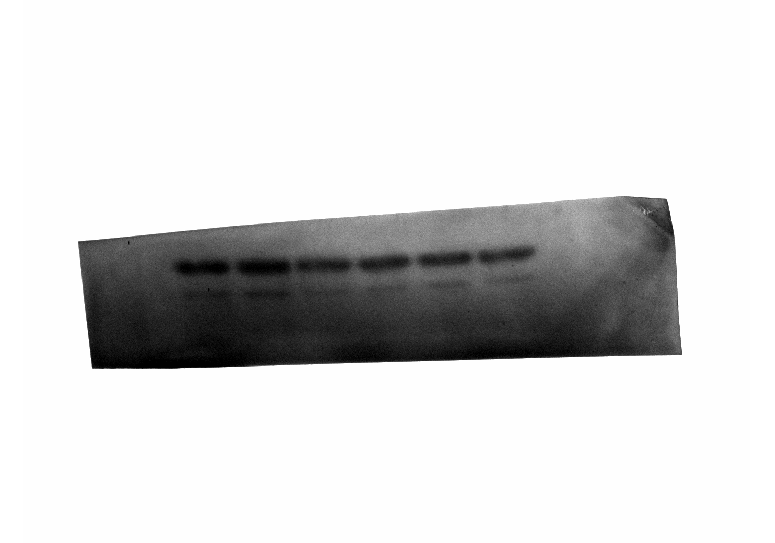

Supplement: Supplemental Information 3 [file peerj-10-12969-s003.zip › Initial data/FIG 1/Time LC3B.tif]

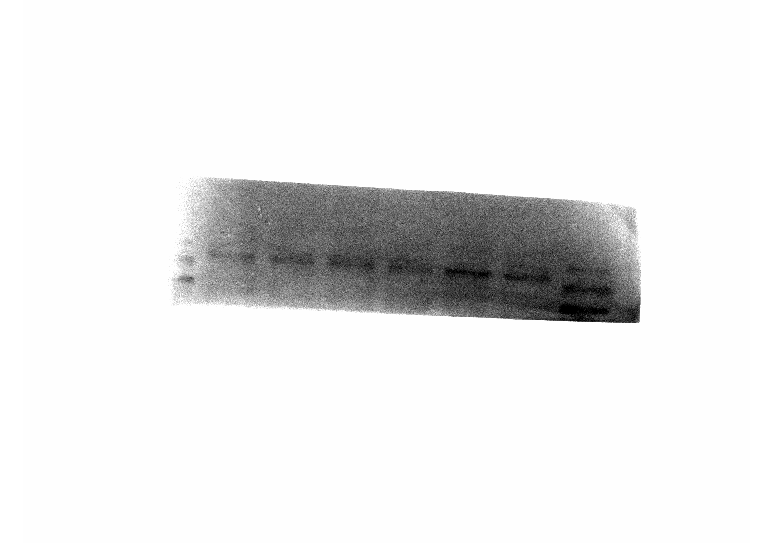

Supplement: Supplemental Information 3 [file peerj-10-12969-s003.zip › Initial data/FIG 1/Time p62.tif]

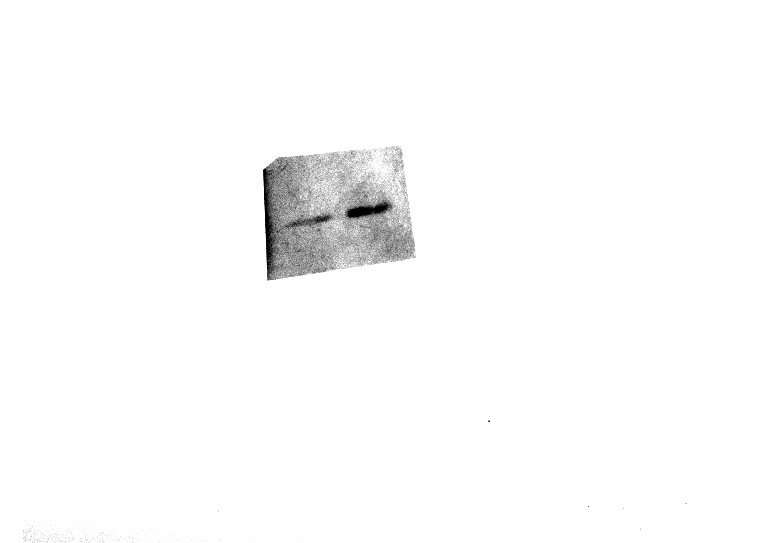

Supplement: Supplemental Information 3 [file peerj-10-12969-s003.zip › Initial data/FIG 2/Cx43.tif]

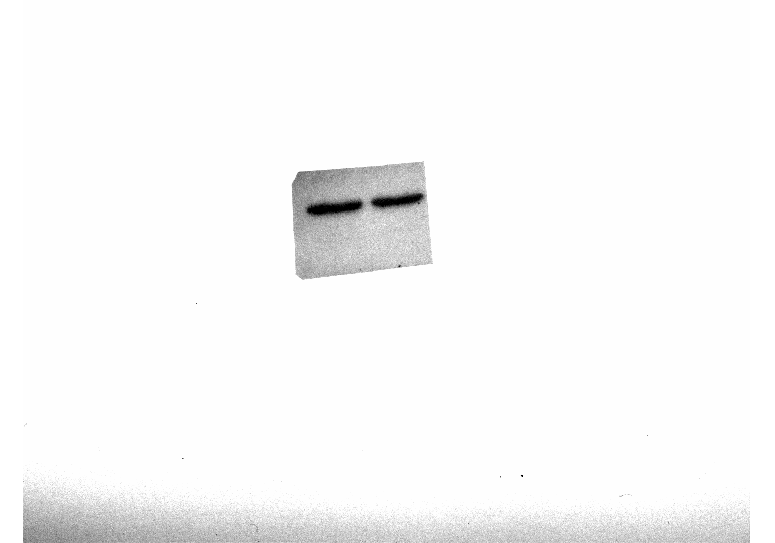

Supplement: Supplemental Information 3 [file peerj-10-12969-s003.zip › Initial data/FIG 2/GAPDH.tif]

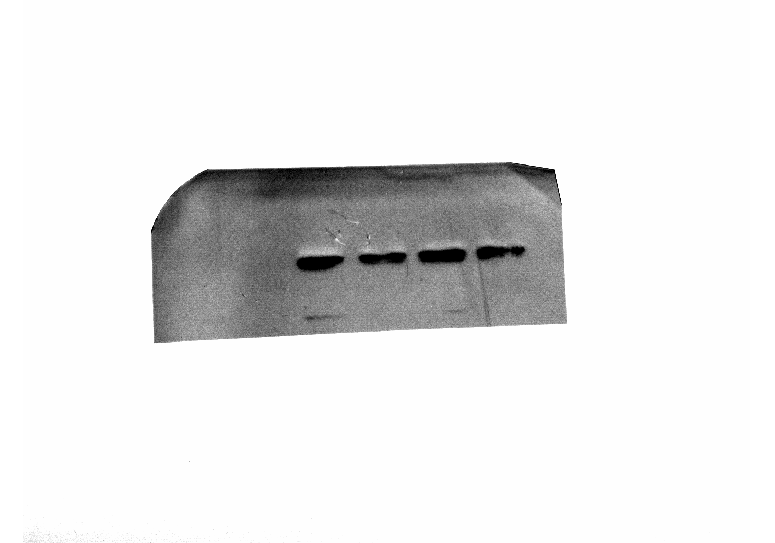

Supplement: Supplemental Information 3 [file peerj-10-12969-s003.zip › Initial data/FIG 3/Beclin 1.tif]

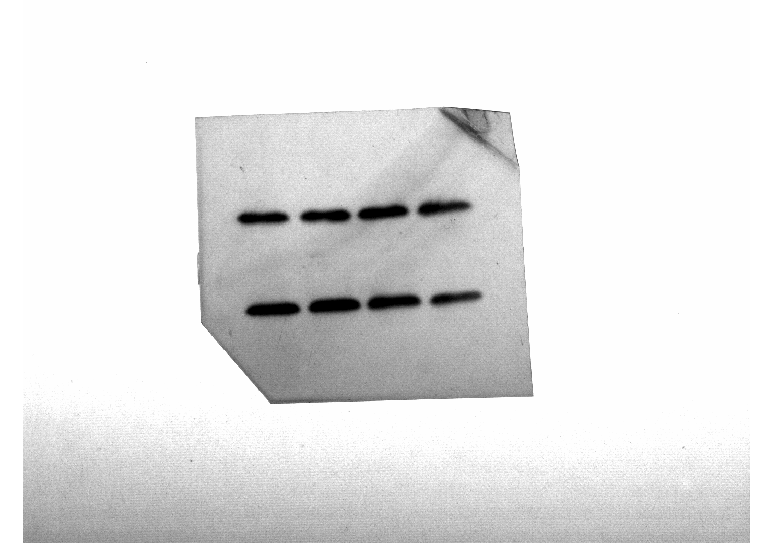

Supplement: Supplemental Information 3 [file peerj-10-12969-s003.zip › Initial data/FIG 3/GAPDH.tif]

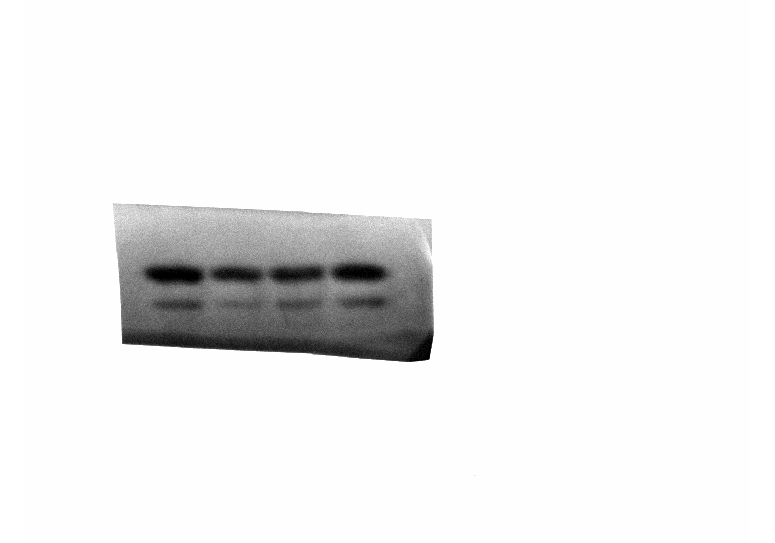

Supplement: Supplemental Information 3 [file peerj-10-12969-s003.zip › Initial data/FIG 3/LC3B.tif]

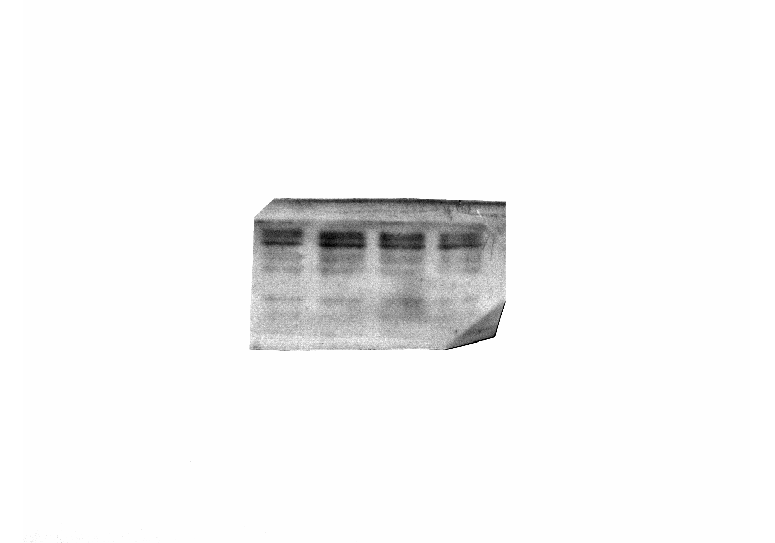

Supplement: Supplemental Information 3 [file peerj-10-12969-s003.zip › Initial data/FIG 3/p62.tif]

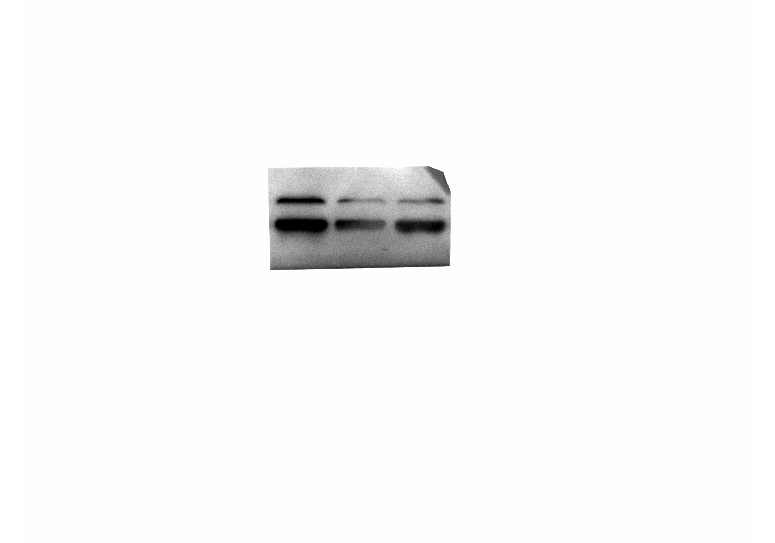

Supplement: Supplemental Information 3 [file peerj-10-12969-s003.zip › Initial data/FIG 4/Beclin 1.tif]

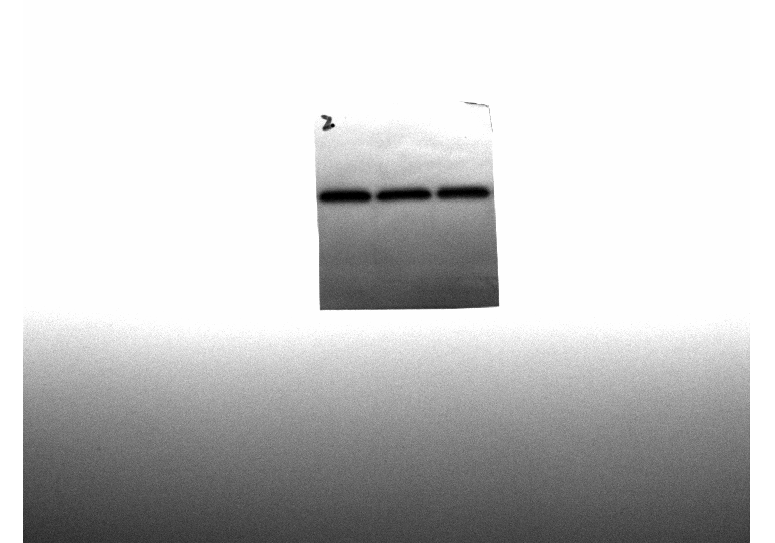

Supplement: Supplemental Information 3 [file peerj-10-12969-s003.zip › Initial data/FIG 4/Cx43-GAPDH.tif]

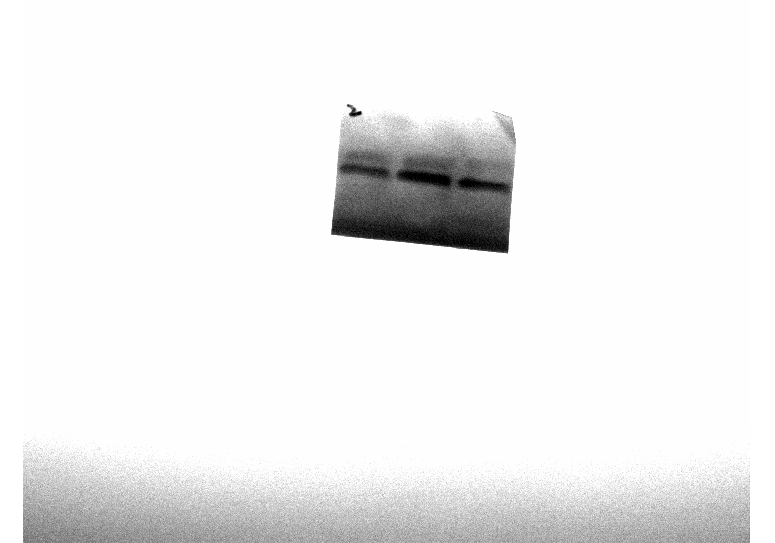

Supplement: Supplemental Information 3 [file peerj-10-12969-s003.zip › Initial data/FIG 4/Cx43.tif]

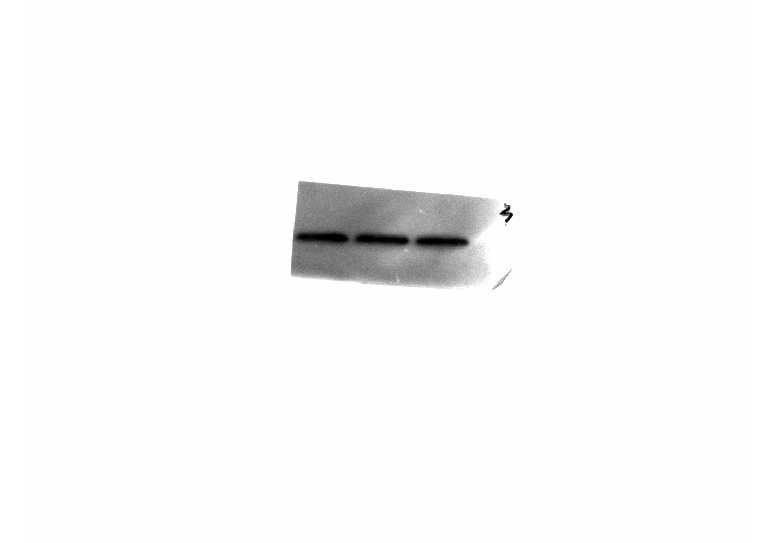

Supplement: Supplemental Information 3 [file peerj-10-12969-s003.zip › Initial data/FIG 4/GAPDH.tif]

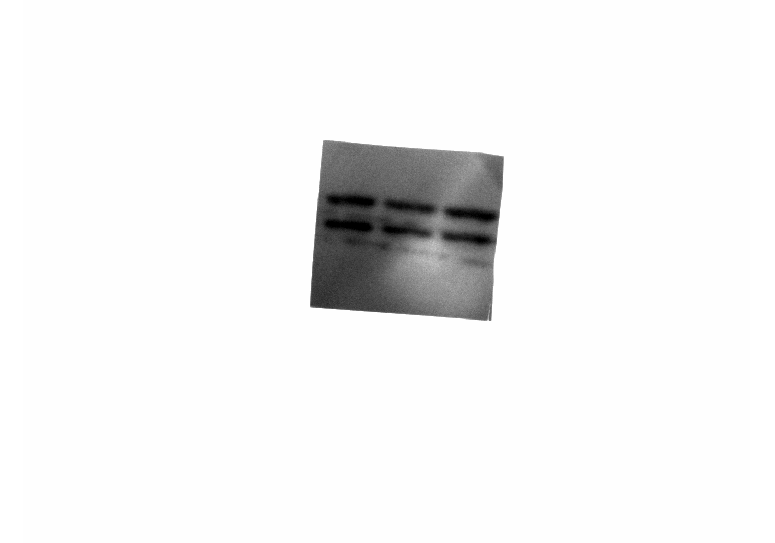

Supplement: Supplemental Information 3 [file peerj-10-12969-s003.zip › Initial data/FIG 4/LC3.tif]

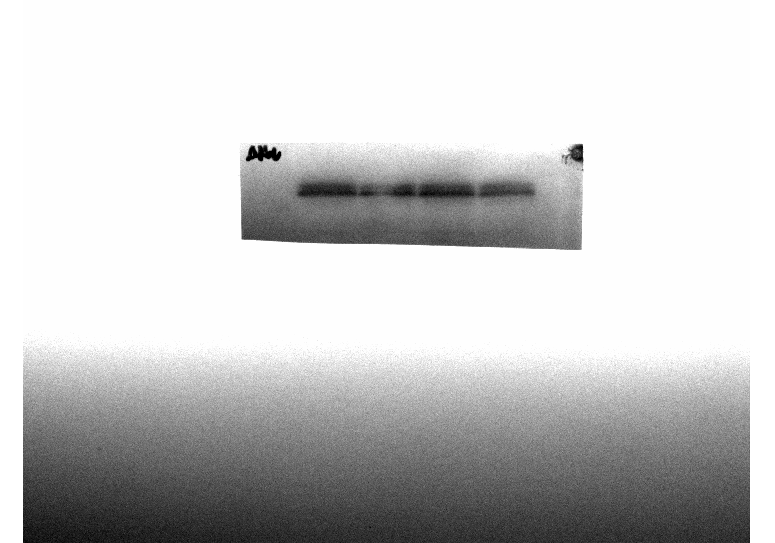

Supplement: Supplemental Information 3 [file peerj-10-12969-s003.zip › Initial data/FIG 4/PI3K AKT mTOR/AKT.tif]

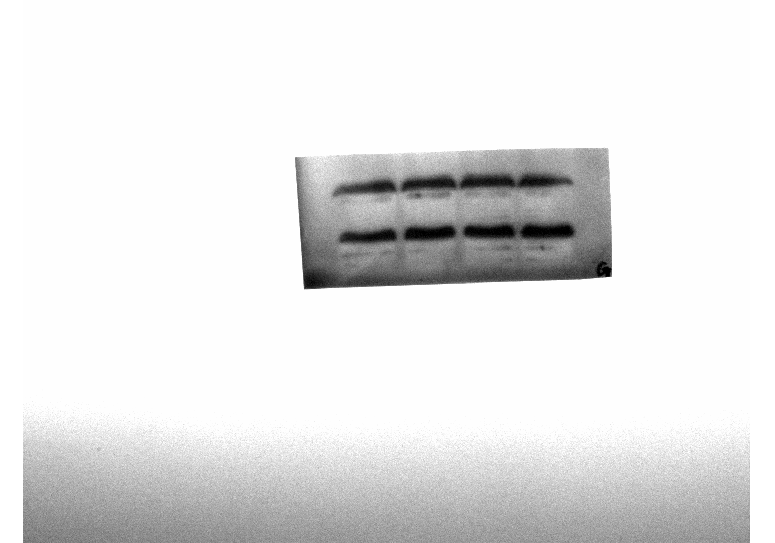

Supplement: Supplemental Information 3 [file peerj-10-12969-s003.zip › Initial data/FIG 4/PI3K AKT mTOR/PI3K.tif]

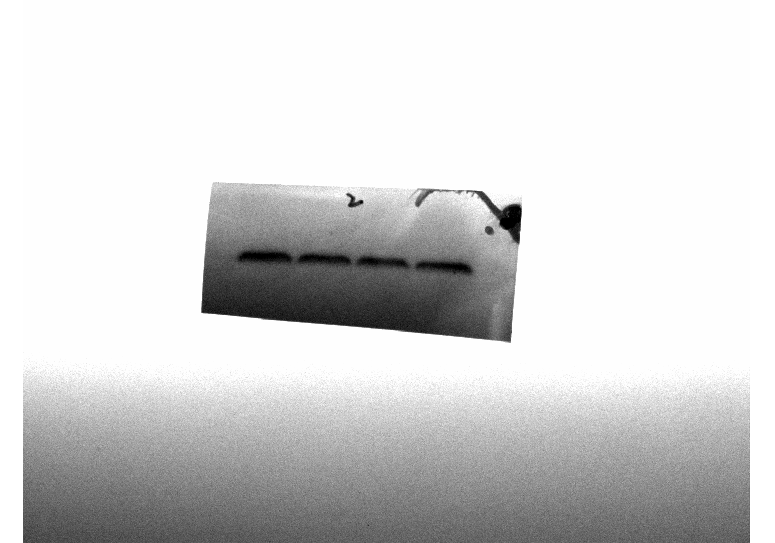

Supplement: Supplemental Information 3 [file peerj-10-12969-s003.zip › Initial data/FIG 4/PI3K AKT mTOR/Tublin.tif]

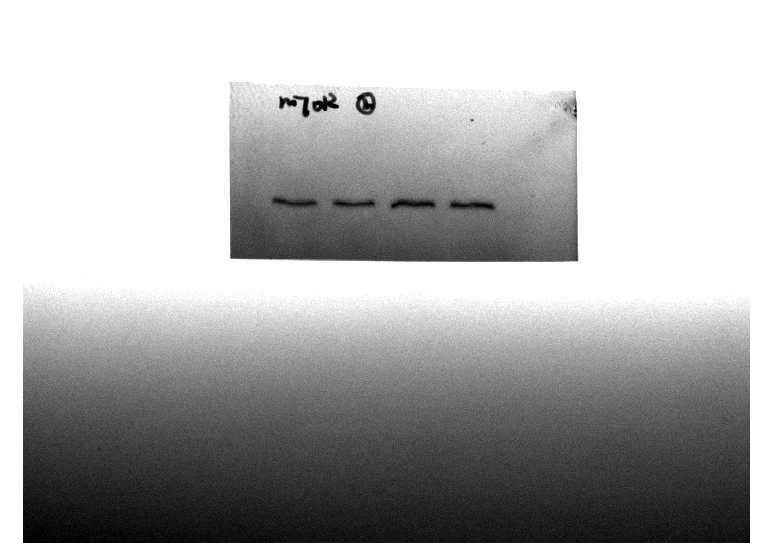

Supplement: Supplemental Information 3 [file peerj-10-12969-s003.zip › Initial data/FIG 4/PI3K AKT mTOR/mTOR.tif]

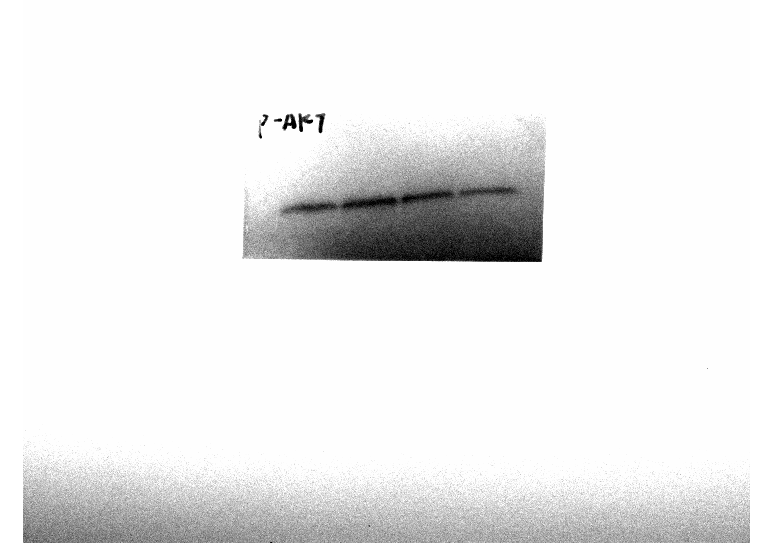

Supplement: Supplemental Information 3 [file peerj-10-12969-s003.zip › Initial data/FIG 4/PI3K AKT mTOR/p-AKT.tif]

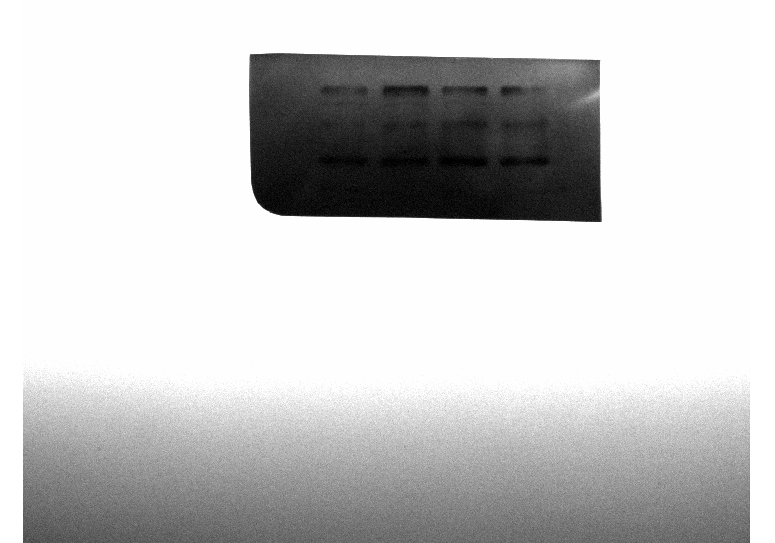

Supplement: Supplemental Information 3 [file peerj-10-12969-s003.zip › Initial data/FIG 4/PI3K AKT mTOR/p-PI3K.tif]

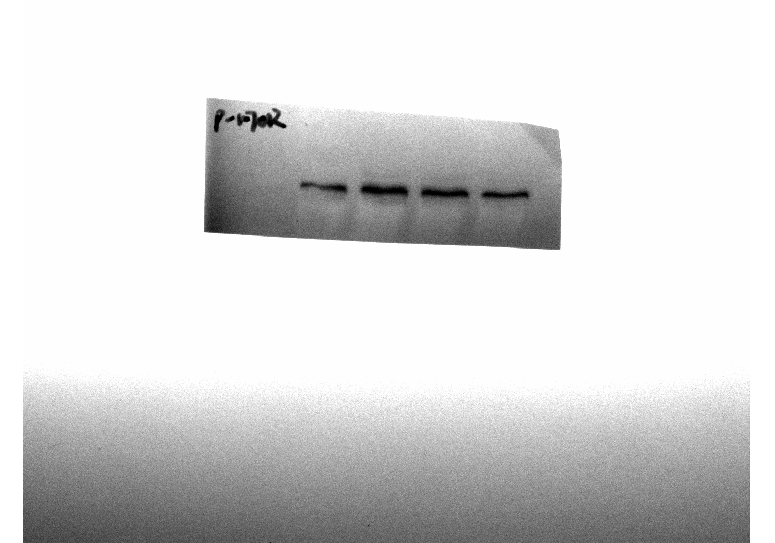

Supplement: Supplemental Information 3 [file peerj-10-12969-s003.zip › Initial data/FIG 4/PI3K AKT mTOR/p-mTOR.tif]

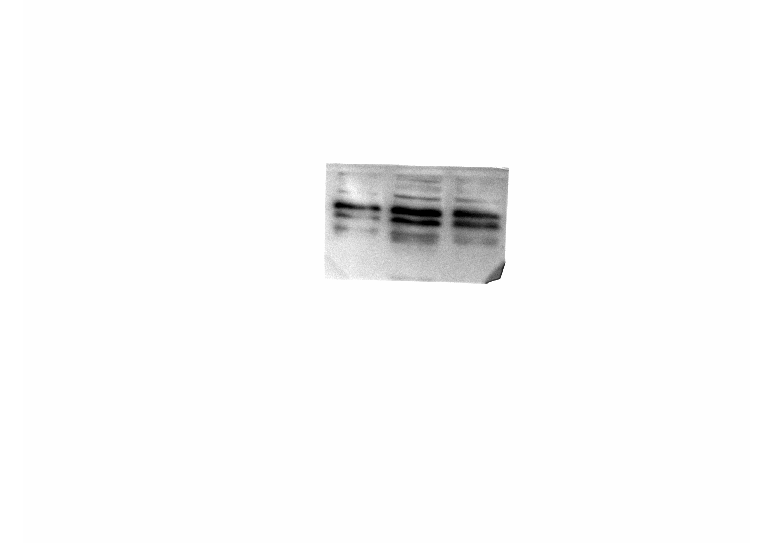

Supplement: Supplemental Information 3 [file peerj-10-12969-s003.zip › Initial data/FIG 4/p62.tif]

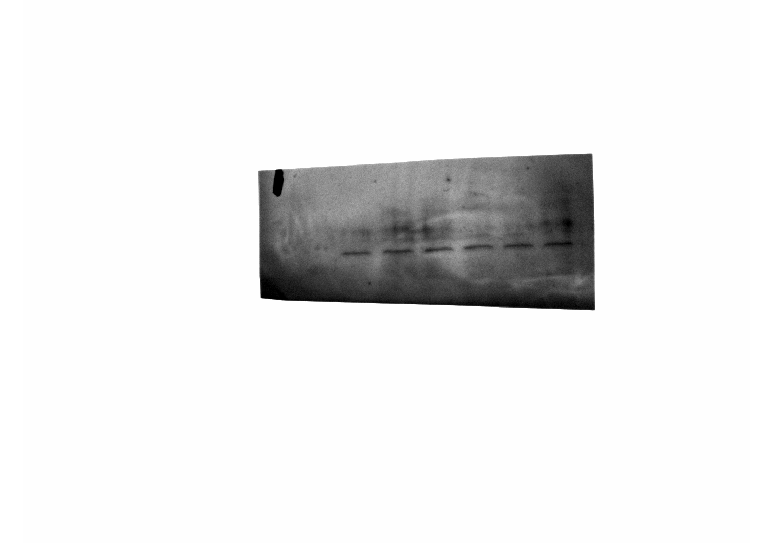

Supplement: Supplemental Information 3 [file peerj-10-12969-s003.zip › Initial data/Supplementary FIG 1/Desmin.tif]

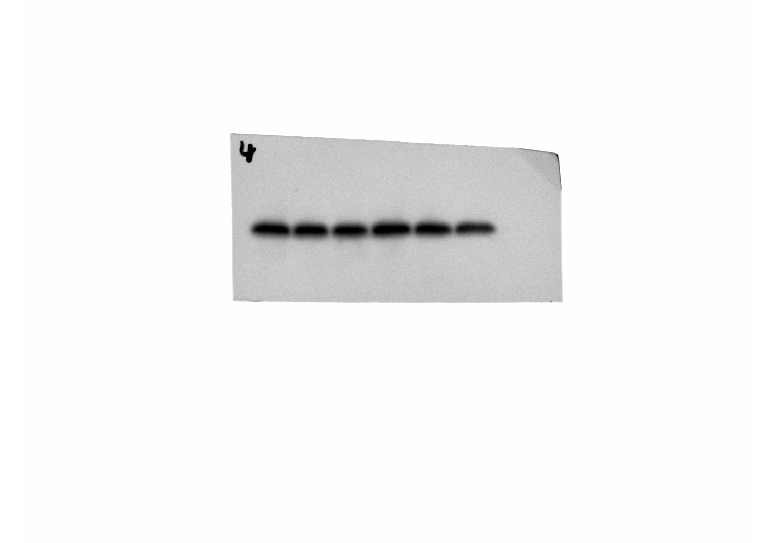

Supplement: Supplemental Information 3 [file peerj-10-12969-s003.zip › Initial data/Supplementary FIG 1/GAPDH.tif]

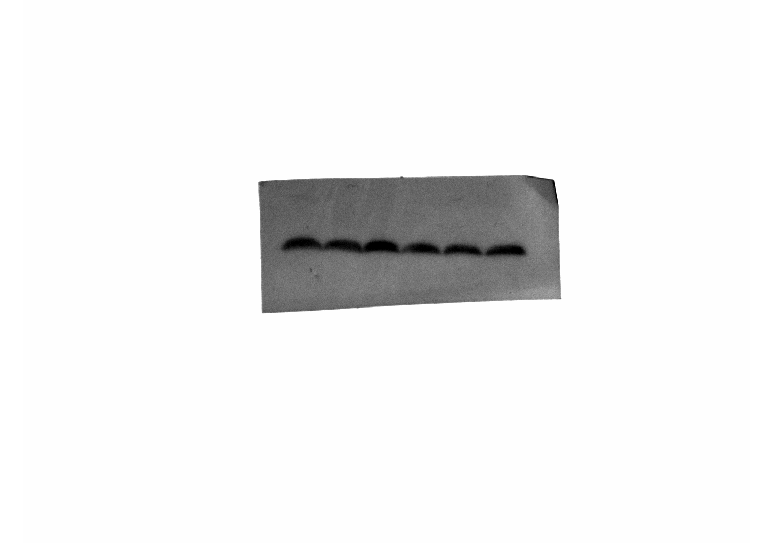

Supplement: Supplemental Information 3 [file peerj-10-12969-s003.zip › Initial data/Supplementary FIG 1/a┴-SMA.tif]
